# Adipose-derived mesenchymal stromal cell-derived exosomes promote tendon healing by activating both SMAD1/5/9 and SMAD2/3

**DOI:** 10.1186/s13287-021-02410-w

**Published:** 2021-06-10

**Authors:** Hengchen Liu, Mingzhao Zhang, Manyu Shi, Tingting Zhang, Wenjun Lu, Shulong Yang, Qingbo Cui, Zhaozhu Li

**Affiliations:** Department of Pediatric Surgery, The Second Affiliated Hospital of Harbin Medical University, No. 246, Xuefu Road, Nangang District, Harbin, 150001 China

**Keywords:** Mesenchymal stromal cells, Tendon stem cells, Exosomes, SMAD, Tendon healing

## Abstract

**Background:**

The use of adipose-derived mesenchymal stromal cell-derived exosomes (ADSC-Exos) may become a new therapeutic method in biomedicine owing to their important role in regenerative medicine. However, the role of ADSC-Exos in tendon repair has not yet been evaluated. Therefore, we aimed to clarify the healing effects of ADSC-Exos on tendon injury.

**Methods:**

The adipose-derived mesenchymal stromal cells (ADSCs) and tendon stem cells (TSCs) were isolated from the subcutaneous fat and tendon tissues of Sprague-Dawley rats, respectively, and exosomes were isolated from ADSCs. The proliferation and migration of TSCs induced by ADSC-Exos were analyzed by EdU, cell scratch, and transwell assays. We used western blot to analyze the tenogenic differentiation of TSCs and the role of the SMAD signaling pathways. Then, we explored a new treatment method for tendon injury, combining exosome therapy with local targeting using a biohydrogel. Immunofluorescence and immunohistochemistry were used to detect the expression of inflammatory and tenogenic differentiation after tendon injury, respectively. The quality of tendon healing was evaluated by hematoxylin-eosin (H&E) staining and biomechanical testing.

**Results:**

ADSC-Exos could be absorbed by TSCs and promoted the proliferation, migration, and tenogenic differentiation of these cells. This effect may have depended on the activation of the SMAD2/3 and SMAD1/5/9 pathways. Furthermore, ADSC-Exos inhibited the early inflammatory reaction and promoted tendon healing in vivo.

**Conclusions:**

Overall, we demonstrated that ADSC-Exos contributed to tendon regeneration and provided proof of concept of a new approach for treating tendon injuries.

**Supplementary Information:**

The online version contains supplementary material available at 10.1186/s13287-021-02410-w.

## Introduction

Tendon is a dense connective tissue consisting of limited tendon cells and abundant extracellular matrix (ECM). Tendon injuries are of significant concern worldwide, with more than 30 million affected patients annually [1]. Tendon healing is slow as a result of its hypocellularity and hypovascularity and involves three overlapping phases: inflammation, proliferation, and remodeling [2, 3]. Furthermore, the self-healing potential of any tissue depends, in part, on its endogenous resident stem cells. The viability and tenogenic differentiation of tendon stem cells (TSCs) are the main mechanisms of tendon repair [4]. However, inflammation during the healing phase may compromise biomechanical function [5–7]. Therefore, it is important to enhance tendon healing by promoting anti-inflammation and the proliferation of TSCs.

Mesenchymal stromal cells have demonstrated great potential in tissue healing [8]. Specifically, adipose-derived mesenchymal stromal cells (ADSCs) are highly beneficial for clinical applications because of their abundant and conveniently accessible sources [9]. When transplanted, ADSCs are able to modulate the inflammatory environment and abundant extracellular matrix (ECM) balance to stimulate tendon regeneration [10–12]. Recent studies have demonstrated that the effectiveness of ADSCs in regenerative medicine is due to their paracrine effects [13]. Thus, ADSCs have been identified as new therapeutic agents in biomedicine [14].

Exosomes are membrane-bound extracellular vesicles that target cells by endocytosis, membrane fusion, or receptor-ligand interaction and are important paracrine factors for stromal cells [15]. In addition, exosomes play important roles in immune regulation, apoptosis, and tissue regeneration [16]. The therapeutic effect of ADSC-Exos has been demonstrated in multiple diseases. This is of great significance in the future development of tissue repair and regeneration engineering [17].

We hypothesize that ADSC-Exos promote tendon repair by regulating the biological characteristics of TSCs as well as the extracellular microenvironment. Specifically, in this study, we investigated the effects of ADSC-Exos on the proliferation, migration, and differentiation of TSCs in vitro, and during inflammation and regeneration situations in vivo.

## Materials and methods

### Animals

Male Sprague-Dawley rats weighing 180–230 g at 8–10 weeks of age were provided by the Experiment Center of Harbin Medical University (Harbin, Heilongjiang, China). All animals were treated according to the United States National Institutes of Health Guide for the Care and Use of Laboratory Animals, and the protocol was approved by the corresponding ethics committee (no. Ky2018-135).

### Isolation and identification of TSCs and ADSCs

The isolation methods of ADSCs and TSCs were performed as in previous studies [18, 19]. In brief, TSCs were isolated from rat tendon and cultured in Dulbecco’s modified Eagle’s medium (DMEM) (Invitrogen, Carlsbad, CA, USA) containing 10% fetal bovine serum (FBS) (Biological Industries, Kibbutz Beit-Haemek, Israel) and 1% penicillin-streptomycin (Beyotime, Haimen, China). The multilineage differentiation potential of TSCs, as well as the identification of surface markers (CD90- and CD105-positive; CD106- and CD11b-negative), was demonstrated in our previous study [19]. ADSCs were isolated from the subcutaneous fat of rats and cultured in DMEM/F12 (Invitrogen) containing 10% FBS and 1% penicillin-streptomycin. Flow cytometry was used to identify surface markers. The adipogenic, osteogenic, and chondrogenic differentiation of ADSCs was induced in a differentiation medium (Cyagen, Santa Clara, CA, USA) to identify their differentiation potential.

### Isolation and identification of ADSC-Exos

At 80% confluence, the culture medium of the ADSCs was changed to exosome-depleted medium (DMEM/F12 containing 10% exosome-depleted FBS (Biological Industries) and 1% penicillin-streptomycin) and incubated for 24 h. Then, the culture medium was collected without ADSCs and centrifuged at 300×*g* for 10 min, 3000×*g* for 10 min, 10,000×*g* for 30 min, and 100,000×*g* for 2 h to isolate the exosomes. Exosomes attached to the bottom of the centrifuge tube were diluted with phosphate-buffered saline. Nanoparticle tracking analysis (NTA), transmission electron microscopy (TEM), and western blotting were used to identify and evaluate the collected exosomes.

### Cellular internalization of ADSC-Exos

ADSC-Exos were incubated with 1 μM PKH26 (Sigma-Aldrich, St. Louis, MO, USA) in Diluent C (Sigma-Aldrich) for 5 min, and excess dye was removed by ultracentrifugation. The labeled exosomes were subsequently added to the serum-free medium of TSC cultures and incubated overnight. The nuclei were labeled with Hoechst 33342 (UE, China), and photos were taken with an inverted fluorescence microscope (Leica, Wetzlar, Germany).

### ADSC-Exo release analysis

The ADSC-Exo release analysis was performed using the BCA protein assay kit (Beyotime, China) as previously described [20]. Briefly, gelatin methacryloyl (GelMA) loaded with 200 μg ADSC-Exos was immersed in PBS in a 24-well plate. The supernatant was collected every 24 h for determining ADSC-Exo release, and new PBS was added. The released ADSC-Exos were quantified and expressed as the release percentage.

### Treatment of TSCs with ADSC-Exos

First, to determine the effect of ADSC-Exo treatment on TSCs, 1 × 10^6^ TSCs were seeded into six-well culture plates for 24 h and divided randomly into four groups. ADSC-Exos were added to the exosome-free medium at 0, 25, 50, or 100 μg/mL and used to replace the TSC culture medium. Next, to further study the related mechanisms, we randomly sorted TSCs seeded in six-well culture plates into four groups as follows: (1) control: exosome-free medium was used to replace the TSC culture medium; (2) ADSC-Exos: 50 μg/mL ADSC-Exos was added to the exosome-free medium and used to replace the TSC culture medium; (3) ADSC-Exos+SB: 10 nM of the SMAD2/3 inhibitor SB431542 (MedChemExpress, Monmouth Junction, NJ, USA) was added to the TSCs 30 min before the addition of 50 μg/mL ADSC-Exos; and (4) ADSC-Exos+DM: 10 nM of the SMAD1/5/9 inhibitor dorsomorphin (MedChemExpress) was added to the TSCs 30 min before addition of 50 μg/mL ADSC-Exos. TSCs from all the experimental groups were collected after 30 min or 24 h for western blotting. In addition, EdU, scratch, and transwell assays were performed after 24 h.

### EdU assay

For the cell proliferation analysis, TSCs were incubated with 50 μM 5-ethynyl-2′-deoxyuridine (EdU) from an EdU Assay Kit (UE) for 4 h. The TSCs were then fixed with 4% paraformaldehyde and stained using the same EdU assay kit. The nuclei were labeled with Hoechst 33342, and photos were taken with an inverted fluorescence microscope.

### Scratch assay

TSCs at 2 × 10^5^ cells/well were inoculated into a 6-well plate for overnight culture. A straight-line wound was made in the cultured cells using a sterile 200-μL pipette tip. A serum-free medium with ADSC-Exos was then added into each well. Images were obtained at 0 and 24 h after ADSC-Exo treatment using an inverted microscope with an Axiocam 506 camera and ZEN 2011 software (Zeiss, Oberkochen, Germany).

### Transwell assay

TSCs at 1 × 10^5^ cells/well were inoculated into the transwell upper chamber, and ADSC-Exos were added into the lower compartment. After culturing for 24 h, the TSCs were fixed with absolute ethanol, then stained with crystal violet. Images were obtained under a light microscope.

### Western blot analyses

TSCs were lysed in radioimmunoprecipitation assay buffer (Beyotime). Immunoblotting was performed using the following rabbit primary antibodies: anti-CD9 (monoclonal; 1:2000; ab92726; Abcam, Cambridge, UK), abti-TSG101 (monoclonal; 1:2000; ab125011; Abcam), anti-Hsp70 (monoclonal; 1:1000; ab2787; Abcam), anti-tenomodulin (anti-TNMD; polyclonal; 1:1000; ab203676; Abcam), anti-collagen I (monoclonal; 1:1000; ab270993; Abcam), anti-scleraxis (anti-SCXA; polyclonal; 1:500; DF13293; Affinity Biologicals, Ancaster, ON, Canada), anti-alkaline phosphatase (anti-ALP; polyclonal; 1:1000; DF6225; Affinity Biologicals), anti-runt-related transcription factor 2 (anti-Runx2; monoclonal; 1:1000; ab264077; Abcam), anti-SMAD2/3 (monoclonal; 1:1000; 5678S; Cell Signaling Technology, Danvers, MA, USA), anti-phospho (p)-SMAD2/3 (monoclonal; 1:1000; 8828S; Cell Signaling Technology), anti-SMAD1/5/9 (polyclonal; 1:500; AF0614; Affinity Biologicals), anti-phospho-SMAD1/5/9 (polyclonal; 1:1000; AF8313; Affinity Biologicals), and anti-β-actin (monoclonal; 1:5000; ab8226; Abcam). Horseradish peroxidase-conjugated goat anti-rabbit IgG (1:5000; BA1055; Boster, Wuhan, China) was used as the secondary antibody. A chemiluminescence imaging system (ChemiScope 6200T, Clinx Science Instruments, Shanghai, China) was used for detection.

### Experimental protocols and surgical procedures

A total of 63 Sprague-Dawley rats were divided into three groups of 21: (1) control: animals that underwent surgery for partial resection of the patellar tendon; (2) GelMA: animals that underwent surgery for patellar tendon partial resection and were inoculated with 30 μL GelMA (EFL-GM-60, 10% w/v) over the tendon defect; and (3) ADSC-Exos: animals for which the injured patellar tendon was treated with 30 μL GelMA containing 200 μg of ADSC-Exos. The exosome content was determined according to previous studies [19]. Rats were anesthetized with 0.3% sodium pentobarbital (30 mg/kg). The right patellar tendon was surgically exposed, and the central 1/3 of the tendon tissue was removed as in previous studies [21]. GelMA was then inoculated into the lesion and cross-linked into a gel state by ultraviolet light. The skin incision was closed using 4-0 sutures. The modeling process is shown in Figure S1. Animals from each group (*n* = 6) were euthanized on day 7 for immunofluorescence analyses and on days 14 or 28 for immunohistochemical analysis.

### Histopathological and immunohistochemical analyses

Paraffin-embedded tendon tissues were sectioned at a thickness of 4 μm. The tissues were then stained with H&E for histopathological analysis. The stained patellar tendons were evaluated according to a previously described parallel fiber alignment scoring method using light microscopy [22]. The scoring scale was as follows: 0, 0–25% parallel fiber alignment; 1, 25–50% parallel fiber alignment; 2, 50–75% parallel fiber alignment; and 3, 75–100% parallel fiber alignment.

For immunohistochemical analyses, the paraffin sections of tendon tissues were incubated with Immuno-Block reagent for 30 min after being deparaffinized and rehydrated. The sections were then incubated with the rabbit primary antibodies: anti-CD146 (monoclonal; 1:250; ab75769; Abcam), anti-TNMD (polyclonal; 1:100; ab203676; Abcam), anti-collagen I (polyclonal; 1:100; ab270993; Abcam), anti-SCXA (polyclonal; 1:100; DF13293; Affinity), anti-ALP (polyclonal; 1:200; DF6225; Affinity), and anti-Runx2 (monoclonal; 1:1000; ab264077; Abcam). Horseradish peroxidase-conjugated goat anti-rabbit IgG (1:500; 115-035-003; Jackson ImmunoResearch, Ely, UK) was used as the secondary antibody. After counterstaining with hematoxylin, the sections were dehydrated and fixed. The area of the positive signal was determined using the ImageJ software.

For immunofluorescence analyses, the sections of tendon tissues were incubated with the rabbit primary antibodies: anti-CCR7 (monoclonal; 1:200; ab32527; Abcam), anti-CD163 (monoclonal; 1:100; ab182422; Abcam), anti-IL-6 (monoclonal; 1:100; TA500067S; Origene), and anti-IL-10 (monoclonal; 1:100; ab33471; Abcam). The sections were then incubated with secondary antibodies (1:200; SA00013; Proteintech, Rosemount, IL, USA) for 1 h. The nuclei were labeled with 4′,6-diamidino-2-phenylindole, and photos were taken with a DM4 B microscope (Leica). Three fields per section were selected randomly for statistical analysis. Positive signals were quantified with the ImageJ software.

### Biomechanical testing

At week 4, patellar tendon tissues from each group (*n* = 3) were taken, and two bony ends of a healing tendon were fixed on a universal material testing machine (Zwick, Roell, Germany). The tissues were investigated using a standard failure test with a testing speed of 5 mm/min. Failure load (N) and stiffness (N/mm) were obtained by the software of the testing machine. Young’s modulus (N × 10^3^/mm^2^) was calculated after measuring the cross-sectional area (mm^2^) of the tendon with a vernier caliper.

### Statistical analyses

All values are expressed as means ± standard deviation. Quantitative data for each group were analyzed by a one-way analysis of variance followed by the Tukey-Kramer test. *P* < 0.05 was considered statistically significant.

## Results

### Characterization of ADSCs

ADSCs exhibited long fusiform morphology (Figure S2A) and differentiated into adipocytes, osteoblasts, and chondroblasts in vitro (Figure S2B). Flow cytometric analysis of ADSC surface markers revealed that the cells were CD90- and CD105-positive, and CD34-, CD45-, and CD11b-negative (Figure S2C).

### Characterization and internalization of ADSC-Exos

**Fig. 1:**
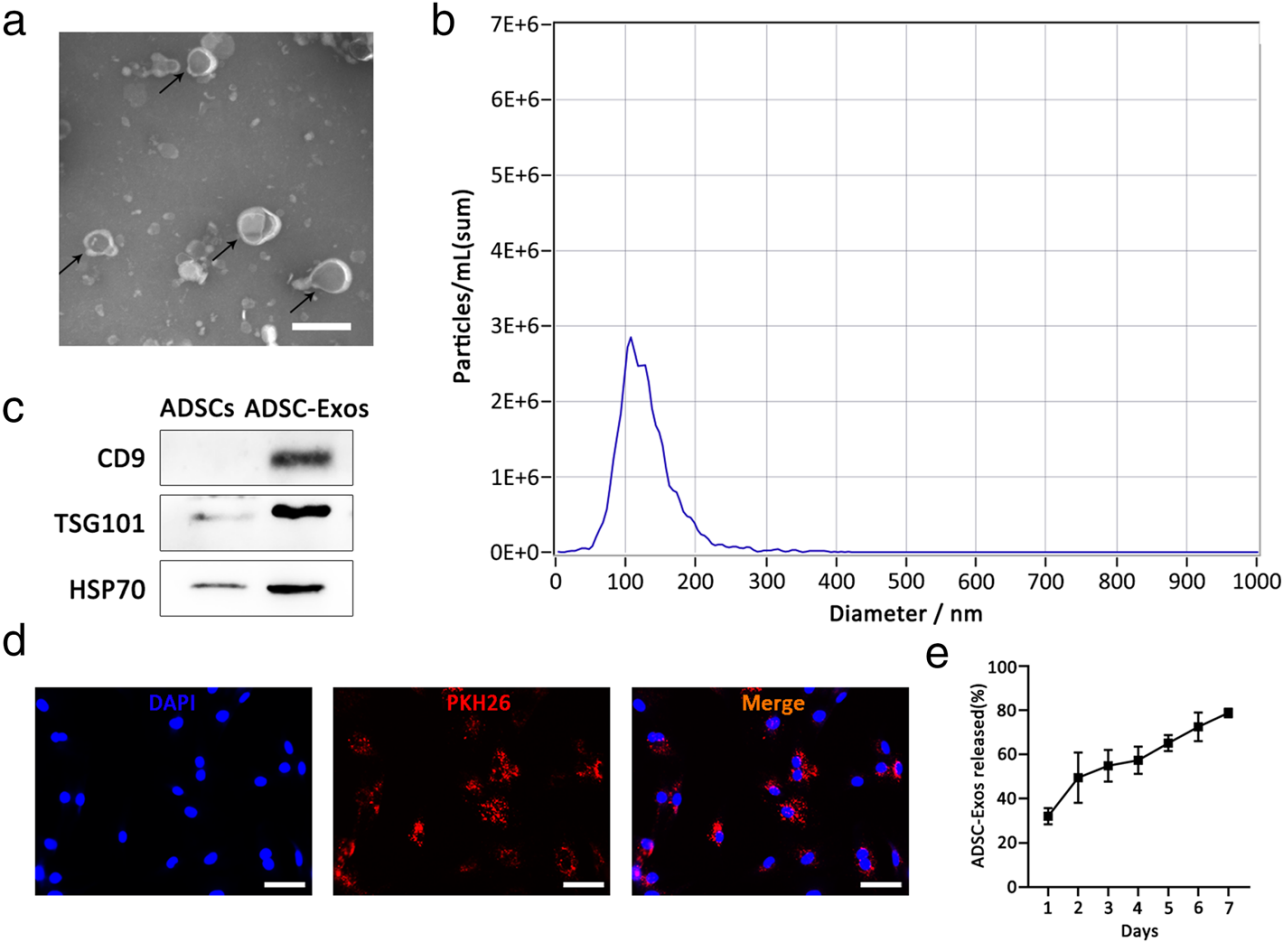
Characterization of ADSC-Exos. **a** Morphology of ADSC-Exos under a transmission electron microscope. **b** Particle size distribution. **c** Western blot was used to detect exosome surface markers. **d** PKH26-labeled ADSC-Exos internalization by TSCs. **e** Profile of ADSC-Exos released from the GelMA. Bars (ADSC-Exos) 100nm; bars (ADSCs), 100μm

TEM revealed that ADSC-Exos were round or elliptical vesicular structures (Fig. 1a). The NTA revealed the mean diameter of ADSC-Exos to be 109.6 nm (Fig. 1b). Western blot analyses confirmed that the ADSC-Exo surface markers CD9, TSG101, and HSP70 were positively expressed (Fig. 1c). In addition, ADSC-Exos were internalized by TSCs and showed red fluorescence (Fig. 1d). Finally, the release behavior of ADSC-Exos loaded in GelMA is shown in Fig. 1e.

### ADSC-Exos promoted the proliferation, migration, and tenogenic differentiation of TSCs

**Fig. 2:**
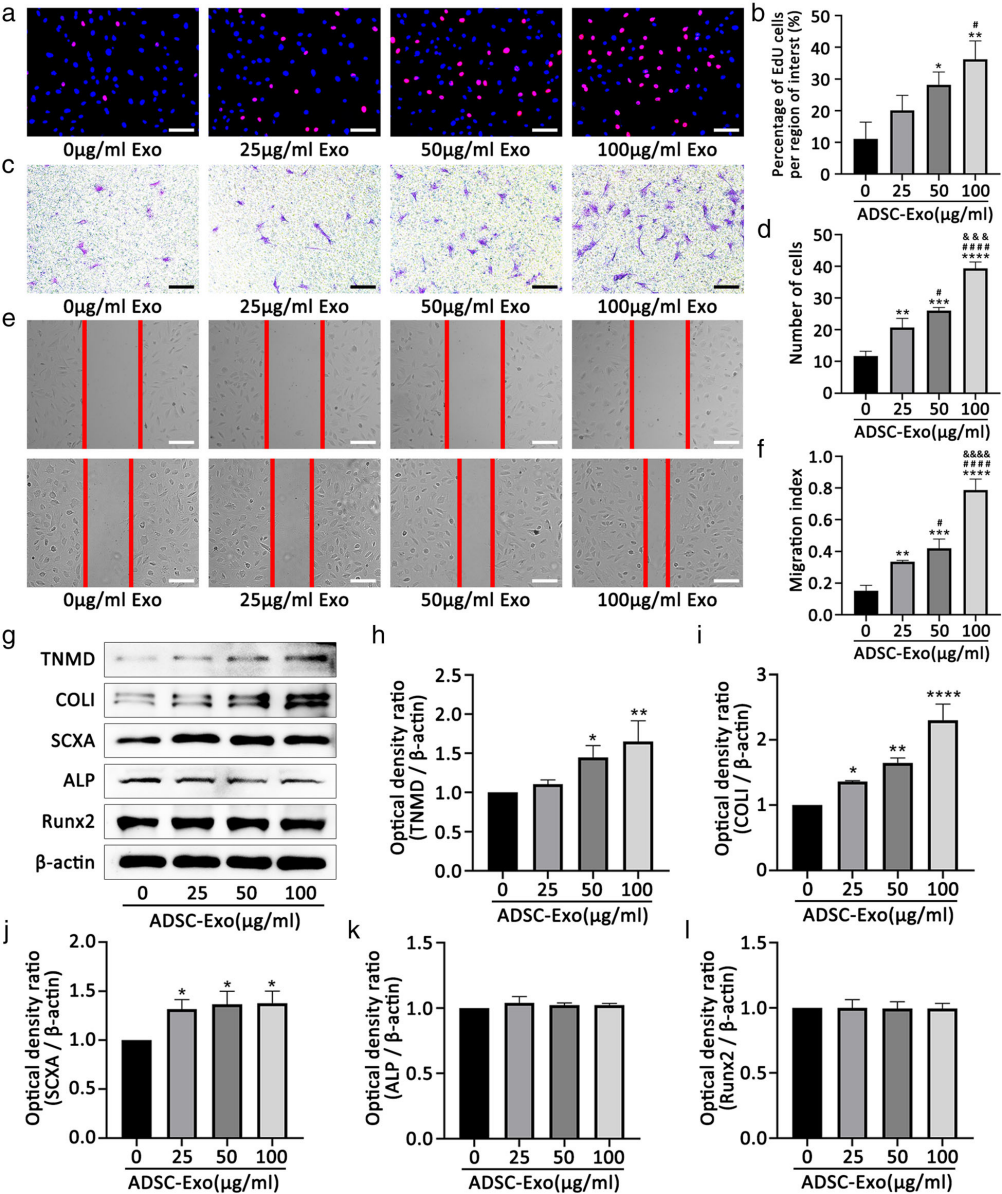
ADSC-Exos promote the proliferation, migration, and tenogenic differentiation of TSCs. **a**, **b** Effect of different concentrations of ADSC-Exos on the proliferation of TSCs by EdU assays. **c–f** Effect of different concentrations of ADSC-Exos on the migration of TSCs by transwell assays and scratch assays. **g–l** Western blot analysis of protein levels of TNMD, collagen I, SCXA, ALP, and Runx2 induced by different concentrations of ADSC-Exos. Bars, 100 μm. Data are represented as mean ± SD. *vs control group; ^#^vs 25 group; ^&^vs 50 group; *n* = 3. *P < 0.05, **P < 0.01, ***P < 0.001, ****P < 0.0001, ^#^*P* < 0.05, ^####^*P* < 0.0001, ^&&&^*P* < 0.001, ^&&&&^*P* < 0.0001

We first measured the effect of the different concentrations of ADSC-Exos on the proliferation and migration of TSCs. The EdU assay showed that ADSC-Exos promoted TSC proliferation (Fig. 2a, B). Further, the transwell assay confirmed that ADSC-Exos promoted TSC migration with increasing concentrations of exosomes (Fig. 2c, d). The scratch test showed results consistent with these findings (Fig. 2e, f). Then, we investigated whether ADSC-Exos affected the differentiation of TSCs. Western blot analyses showed ADSC-Exos significantly increased the protein expression of TNMD, collagen I, and SCXA but had no effect on ALP or Runx2 (Fig. 2g–l). These results suggest that ADSC-Exos promote the tenogenic differentiation ability of TSCs but have no effect on osteogenic differentiation.

### ADSC-Exos activated the SMAD2/3 and SMAD1/5/9 pathways

**Fig. 3:**
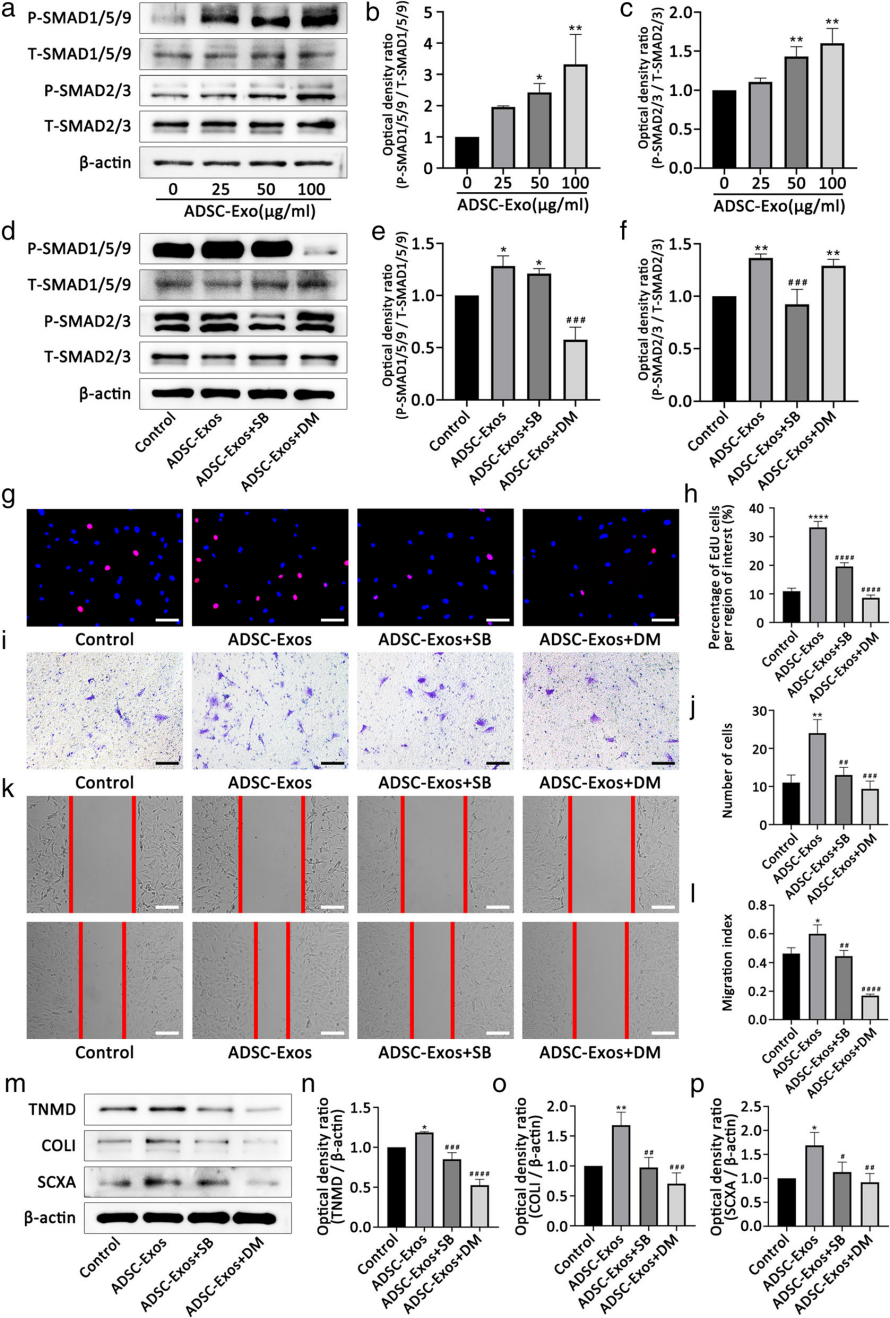
ADSC-Exos promote the proliferation, migration, and tenogenic differentiation of TSCs via the SMAD2/3 and SMAD1/5/9 signaling pathways. **a–c** Western blot analysis of protein levels of p-SMAD2/3 and p-SMAD1/5/9 induced by different concentrations of ADSC-Exos. **d–f** SB431542 and dorsomorphin inhibit the activation of SMAD2/3 and SMAD1/5/9 induced by ADSC-Exos, respectively. **g**, **h** EdU assay showed that ADSC-Exos-mediated TSC proliferation was suppressed by inhibitors SB431542 and dorsomorphin. **i–l** Transwell assay and scratch assays showed that ADSC-Exos-mediated TSC migration was suppressed by inhibitors SB431542 and dorsomorphin. **m–p** Western blot analysis of protein levels of TNMD, collagen I, and SCXA promoted by ADSC-Exos was inhibited by inhibitors SB431542 and dorsomorphin. Bars, 100 μm. Data are represented as mean ± SD. *vs control group; ^#^vs ADSC-Exos group; *n* = 3. *P < 0.05, **P < 0.01, ***P < 0.001, ****P < 0.0001, ^#^*P* < 0.05, ^##^*P* < 0.01, ^###^*P* < 0.001, ^####^*P* < 0.0001

The SMAD signaling pathways play vital roles in regulating stem cell activity. SMAD2/3 and SMAD1/5/9 are two typical SMAD signaling pathways. Therefore, we examined the changes in these two pathways after ADSC-Exo uptake by TSCs. Western blot analyses showed that ADSC-Exos increased the p-SMAD2/3 and p-SMAD1/5/9 expression in TSCs (Fig. 3a–c), suggesting that the uptake of ADSC-Exos by TSCs activated the SMAD2/3 and SMAD1/5/9 signaling pathways. Furthermore, we pretreated TSCs with the SMAD2/3 inhibitor, SB431542, or the SMAD1/5/9 inhibitor, dorsomorphin, for 30 min. Western blot analyses showed that SB431542 and dorsomorphin inhibited the phosphorylation of SMAD2/3 and SMAD1/5/9, respectively (Fig. 3d–f).

### ADSC-Exos regulated TSC proliferation, migration, and tenogenic differentiation by activating SMAD2/3 and SMAD1/5/9 signaling pathways

To investigate the regulatory effect of ADSC-Exos, we evaluated their effects on the proliferation, migration, and tendon differentiation of TSCs by pretreating them with SB431542 or dorsomorphin. As expected, the proliferation (Fig. 3g, h) and migration (Fig. 3i–l) of TSCs were significantly decreased in the ADSC-Exos + SB431542 and ADSC-Exos + dorsomorphin groups compared with that in the ADSC-Exos only group. Similarly, western blot analyses showed that pretreatment with SB431542 or dorsomorphin significantly decreased the expression of the tenogenic differentiation genes, TNMD, collagen I, and SCXA, in TSCs (Fig. 3m–p).

### ADSC-Exos regulated the early inflammatory response during tendon healing

**Fig. 4:**
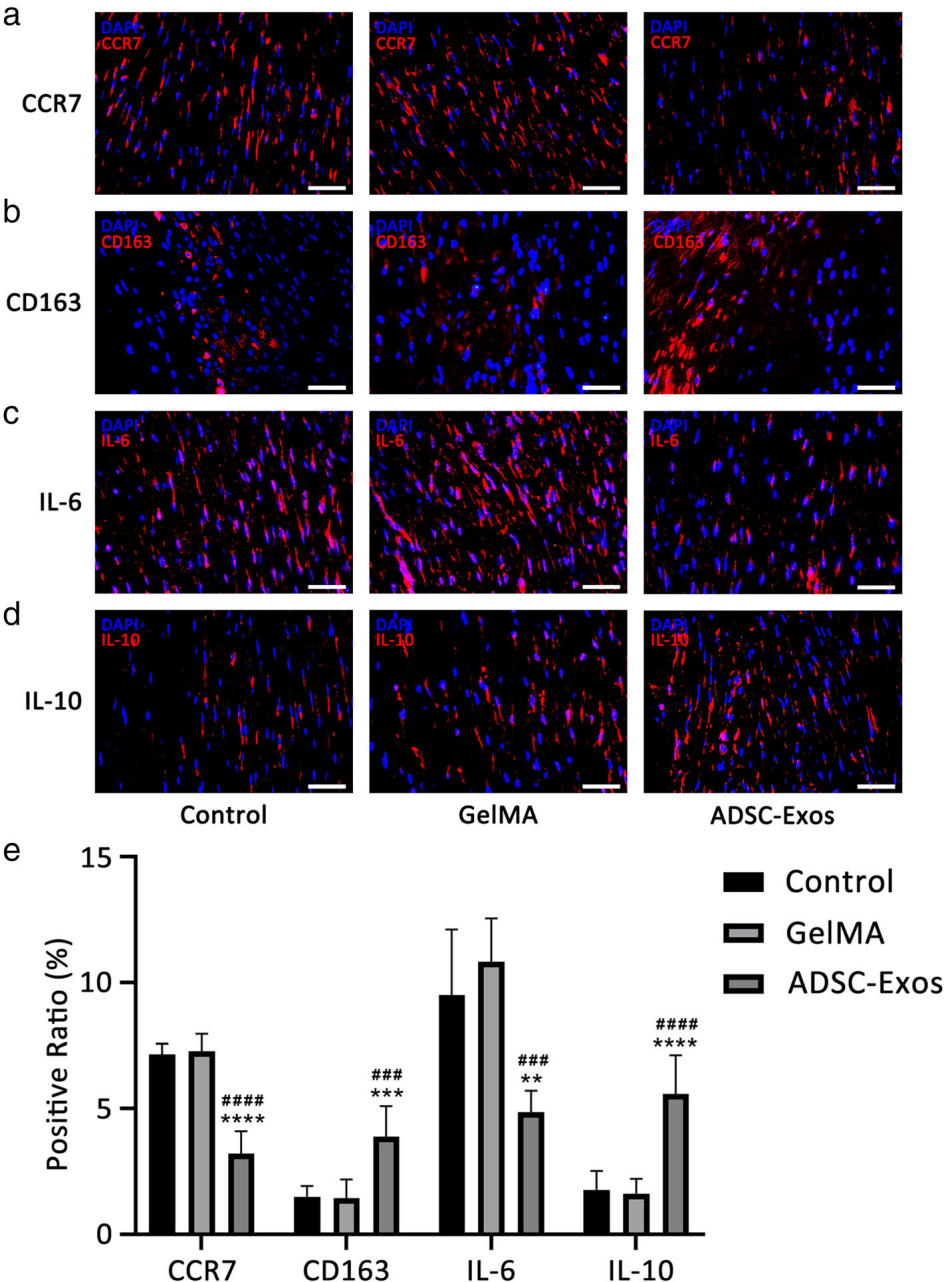
ADSC-Exos inhibits inflammatory expression in tendon injury. **a–d** The expression of CCR7^+^, CD163^+^, IL-6^+^, and IL-10^+^ cells were detected by immunofluorescence at week 1. **e** Positive ratio of inflammation-related factors (*n* = 6). Bars, 50 μm. Data are represented as mean ± SD. ^*^vs control group; ^#^vs GelMA group. **P < 0.01, ***P < 0.001, ****P < 0.0001, ^###^*P* < 0.001, ^####^*P* < 0.0001

We investigated the in vivo effect of ADSC-Exos on early healing of tendon injury. At week 1 after injury, the level of CCR7 (M1 macrophage marker) decreased in the ADSC-Exo group while the level of CD163 (M2 macrophage marker) increased (Fig. 4a, b). Furthermore, IL-10 (an anti-inflammatory factor) increased, and IL-6 (a pro-inflammatory factor) decreased (Fig. 4c, d). Quantitative analyses showed there were more CD163^+^ and IL-10^+^ cells in the ADSC-Exo group, while CCR7^+^ and IL-6^+^ cells predominated in the control and GelMA groups (Fig. 4e).

### ADSC-Exos improved the healing of tendon injury

**Fig. 5:**
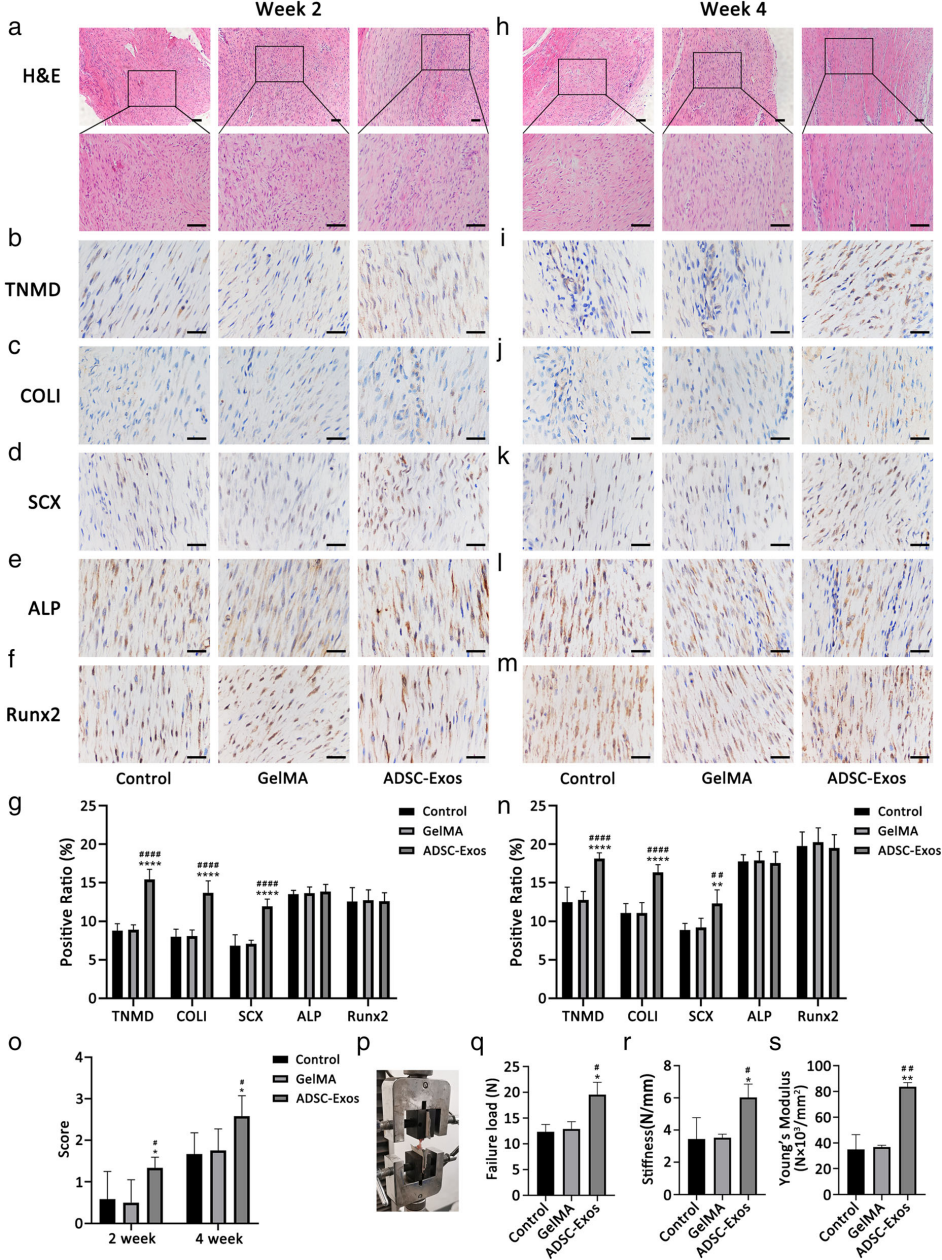
ADSC-Exos improved the healing of tendon injury. **a**, **h** The H&E staining of tendon injury at week 2 and week 4. **b–f**, **i–m** The expression of TNMD, collagen I, SCXA, ALP, and Runx2 were detected by immunohistochemistry assay at week 2 (*n* = 6) and week 4 (*n* = 6). **g**, **n** Quantitative analysis of tenogenic and osteogenic related factors at week 2 (*n* = 6) and week 4 (*n* = 6). **o** Fiber alignment score in each group at week 2 (*n* = 6) and week 4 (*n* = 6). **p–s** Results of biomechanical tests (failure load, stiffness, Young’s modulus) at 4 weeks (*n* = 3). Bars (H&E), 100 μm; bars (immunohistochemistry), 50 μm. Data are represented as mean ± SD. *vs control group; ^#^vs GelMA group; *n* = 6. *P < 0.05, **P < 0.01, ****P < 0.0001, ^#^*P* < 0.05, ^####^*P* < 0.0001

We next assessed whether ADSC-Exos contributed to the healing of patellar tendon injury in rats. H&E staining showed the ADSC-Exo group had much more regular alignment of the fibrous tissue in the defect area at week 2 compared with the other groups (Fig. 5a, o). At week 4, the collagen fiber alignment in the ADSC-Exo group was more compact than in the other groups (Fig. 5h).

Immunohistochemical analyses showed higher expression of TNMD, collagen I, and SCXA in the ADSC-Exo group at week 2 than in the control and GelMA groups (Fig. 5b–d). At week 4, the expression of these three genes remained high in the ADSC-Exo group (Fig. 5i–k). Furthermore, ALP and Runx2 expression were unchanged among the three groups at both weeks 2 and 4 (Fig. 5e, f, l, m). The results of the quantitative analyses are shown in Fig. 5g, n.

Biomechanical testing showed that the failure load, stiffness, and Young’s modulus of the patellar tendon in the ADSC-Exo group were significantly increased compared with the control and GelMA groups (Fig. 5p–s).

### ADSC-Exos promoted TSC proliferation during tendon healing

**Fig. 6:**
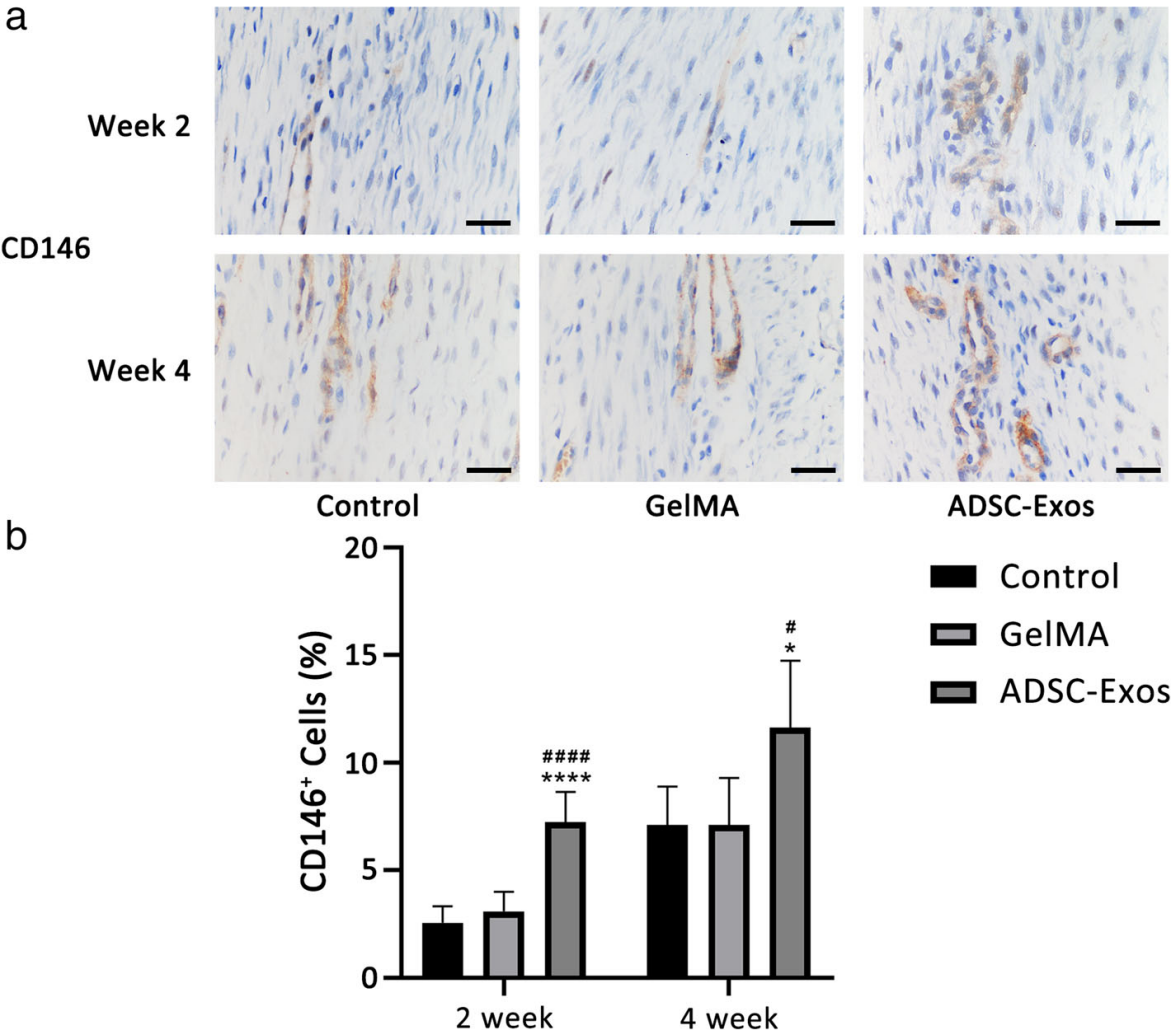
ADSC-Exos promoted TSC proliferation in vivo. **a** Cellular expression of CD146^+^ at week 2 and week 4 was evaluated by immunohistochemistry assay. **b** Ratio of CD146^+^ cells at week 2 (*n* = 6) and week 4 (*n* = 6). Bars, 50 μm. *vs control group; ^#^vs GelMA group; *n* = 6, *P < 0.05, ****P < 0.0001, ^#^*P* < 0.05, ^####^*P* < 0.0001

To investigate the mechanism by which ADSC-Exos promoted tendon healing in vivo, we measured the number of TSCs in the tendon tissue during early healing. CD146 was used as a marker of TSCs [23]. Immunohistochemical staining showed that the number of CD146^+^ TSCs in the injured tendon increased with extension of the healing time. Meanwhile, as expected, the number of CD146^+^ TSCs increased significantly in the ADSC-Exo group (Fig. 6a, b).

## Discussion

Improving the quality of healing after tendon injury remains a major medical challenge. TSCs play an important role in tendon healing [24]. However, Zhang et al. reported that culture-expanded TSCs were prone to lose their phenotypic characteristics and exhibited reduced regeneration ability [25]. Therefore, activating the proliferation and differentiation of TSCs is key to improving tendon healing.

We first studied the influence of ADSC-Exos on TSCs in vitro. The results revealed that ADSC-Exos were internalized into TSCs and promoted their proliferation, migration, and tenogenic differentiation. Implantation of TSCs improves tendon healing in rats [26–29], and the activity of TSCs determines the quality of this healing. It is well-known that the SMAD family of the signaling pathways plays important roles in regulating stem cell functions, with two typical SMAD signaling pathways, SMAD2/3 and SMAD1/5/9, having potential significance in regulating the activity of TSCs [30–32]. Accordingly, we hypothesized that ADSC-Exos promoted the proliferation, migration, and tenogenic differentiation of TSCs by activating the SMAD family signaling pathways. As expected, ADSC-Exos increased the phosphorylation of SMAD2/3 and SMAD1/5/9 in TSCs, which was later found to be attenuated by the inhibitors, SB431542 and dorsomorphin, respectively. We also found that the application of these two inhibitors blocked the effects of ADSC-Exos on the activity of TSCs. These results support the hypothesis that ADSC-Exos enhanced the proliferation and migration of TSCs by promoting the activation of the SMAD2/3 and SMAD1/5/9 signaling pathways.

Tenogenic differentiation is a complex process. SCXA is a key molecule in the early development of tendons. It is responsible for the differentiation of TSCs into tenocytes and the positive regulation of TNMD expression [33, 34]. Subsequently, the TNMD gene is necessary for tendon maturation and has a positive effect on the self-renewal of TSCs [35]. In addition, the expression of collagen I determines the strength of tendons [36]. Because abnormal ossification during tendon healing affects normal tendon functions, we hypothesized that ADSC-Exos would be able to promote tenogenic differentiation and inhibit osteogenic differentiation of TSCs. The results showed that, indeed, ADSC-Exos increased TNMD, collagen I, and SCXA expression in TSCs via activation of the SMAD2/3 and SMAD1/5/9 pathways. However, ADSC-Exos did not affect the expression of ALP or Runx2 in TSCs. This suggests that ADSC-Exos could effectively promote tenogenic differentiation of TSCs, but not inhibit osteogenic differentiation.

Scar formation caused by inflammation after tendon injury is a major cause of histological changes affecting tendon healing prognosis [37]. Therefore, inhibiting the early inflammatory response of tendon injury is beneficial to early healing. Recent studies reported that MSCs can elicit immunoregulatory responses by modulating the pro-inflammatory M1 macrophages to anti-inflammatory M2 macrophage polarization, inducing regulatory T cells, and producing anti-inflammatory cytokines [38, 39]. Considering exosomes are the main substances used by MSCs to exert their effectiveness, we hypothesized that exosomes would recapitulate the immunomodulatory effects of their parent cells. In the current study, we found that CD163+ M2 macrophages were increased significantly in the ADSC-Exo group. In addition, the M2-stimulating factor, IL-10, was increased in the ADSC-Exo group. Furthermore, Shen et al. found that ADSC-Exos reduced the early inflammatory response after tendon injury by regulating macrophages, whereas some studies have confirmed that ADSC-Exos were able to modulate macrophages from an M1 to M2 phenotype in vitro [10, 40–43]. Therefore, we suggest that ADSC-Exos can alleviate early inflammation after tendon injury by modulating macrophages.

Tissue integrity is the standard for evaluating the quality of tendon healing. We used the central 1/3 patellar tendon injury rat model to evaluate tendon healing. H&E staining showed that collagen fibers in the ADSC-Exo group were more regular compared to those in the control and GelMA groups. In addition, the biomechanical properties of the tendon tissues in the ADSC-Exo group were significantly improved at 4 weeks. We also investigated the regulatory effect of ADSC-Exos on TSCs in vivo. Immunohistochemical analyses showed that ADSC-Exos promoted the expression of tenogenic differentiation genes in vivo but did not inhibit the expression of osteogenic differentiation genes in the injured area.

In previous reports, CD146 has been used as a surface marker of TSCs; CD146^+^ TSCs switch to an activated state during tendon-injury healing and increase their proliferation, migration, and tenogenic differentiation ability [44]. Our results showed that the expression of CD146^+^ TSCs in the ADSC-Exo group was the highest among the three groups. This indicated that ADSC-Exos promoted the proliferation ability of CD146^+^ TSCs.

Exosomes are generally used to repair tissues by intravenous or local injection. However, due to difficulty in their local retention, exosomes are unable to exert their full biological efficacy. GelMA is a photosensitive biohydrogel with excellent biocompatibility and degradability and is widely used in various tissue engineering applications [45, 46]. GelMA exists in a liquid state at 37°C and becomes cross-linked under ultraviolet light to form a gel state with ECM properties. Because of its mild response to environmental conditions, GelMA has great advantages for use in biomedicine and is expected to be applicable for various clinical treatments [47]. For instance, Aubin et al. attempted to change the proliferative arrangement of different cells using micropatterned GelMA to provide a theoretical basis for constructing functional tissues in vitro [48]. Zou et al. used GelMA to construct biomimetic bone with a trabecular bone structure, and Hu et al. used GelMA microspheres loaded with small extracellular vesicles to promote cartilage regeneration [20, 49]. In the current study, GelMA was used as a carrier of ADSC-Exos to provide a good microenvironment for exosome storage and their gradual absorption. The ADSC-Exo-loaded GelMA attached to the defect of the patellar tendon in a gel-like manner after cross-linking, and it was gradually absorbed by the body. Therefore, ADSC-Exos loaded into GelMA is a promising treatment for tendon injury.

The current study does have some limitations. First, we selected only one time point to analyze phosphorylation in TSCs. Phosphorylation is a continuous process, and the 30-min time point selected may not be optimal to detect TSC phosphorylation. Second, we only evaluated short-term tendon healing. The long-term therapeutic effect of ADSC-Exos on tendon healing (scar formation) requires further study. Third, the optimal dosage of exosomes for tendon repair warrants further study. In addition, as exosomes contain various proteins, mRNA, and miRNA, further exploration is required to determine the specific substance in exosomes that exerts the therapeutic effect.

## Conclusions

Overall, our results showed that ADSC-Exos were absorbed by TSCs and promoted their proliferation, migration, and tenogenic differentiation via the SMAD2/3 and SMAD1/5/9 signaling pathways. In addition, ADSC-Exos alleviated early inflammation and promoted tendon healing. These findings suggest the potential clinical value of ADSC-Exos in treating tendon defects and provide a new approach for the treatment of tendon injuries.

## Supplementary Information

**Additional file 1: Figure S1.** Patellar tendon injury model. **A** Exposure and removal of central 1/3 right patellar tendon tissue. **B** ADSC-Exos-loaded GelMA injected into the location of the patellar tendon defect. **C** Radiation GelMA with 405nm light source for 30s. **D** Photocross-linking under radiation forms a gel state.

**Additional file 2: Figure S2.** Characterization of ADSCs. **A** Morphology of ADSCs. **B** Adipogenic, osteogenic and chondrogenic differentiation of ADSCs. **C** Flow cytometry for detection of ADSC surface markers. Bars, 100μm.

## Data Availability

The datasets used and/or analyzed during the current study are available from the corresponding author on reasonable request.

## Abbreviations

ADSCs: Adipose-derived mesenchymal stromal cells
ADSC-Exos: Adipose-derived mesenchymal stromal cell-derived exosomes
TSCs: Tendon stem cells
ECM: Extracellular matrix
DMEM: Dulbecco’s modified Eagle’s medium
FBS: Fetal bovine serum
NTA: Nanoparticle tracking analysis
TEM: Transmission electron microscopy
EdU: 5-Ethynyl-2′-deoxyuridine
H&E: Hematoxylin-eosin
TNMD: Tenomodulin
SCXA: Scleraxis
ALP: Alkaline phosphatase
Runx2: Runt-related transcription factor 2
GelMA: Gelatin methacryloyl
