# Supplementary figures and images for: Adipose-derived mesenchymal stromal cell-derived exosomes promote tendon healing by activating both SMAD1/5/9 and SMAD2/3
Source: Stem Cell Res Ther. 2021 Jun 10;12:338. doi: 10.1186/s13287-021-02410-w (PMC8194238; doi:10.1186/s13287-021-02410-w)

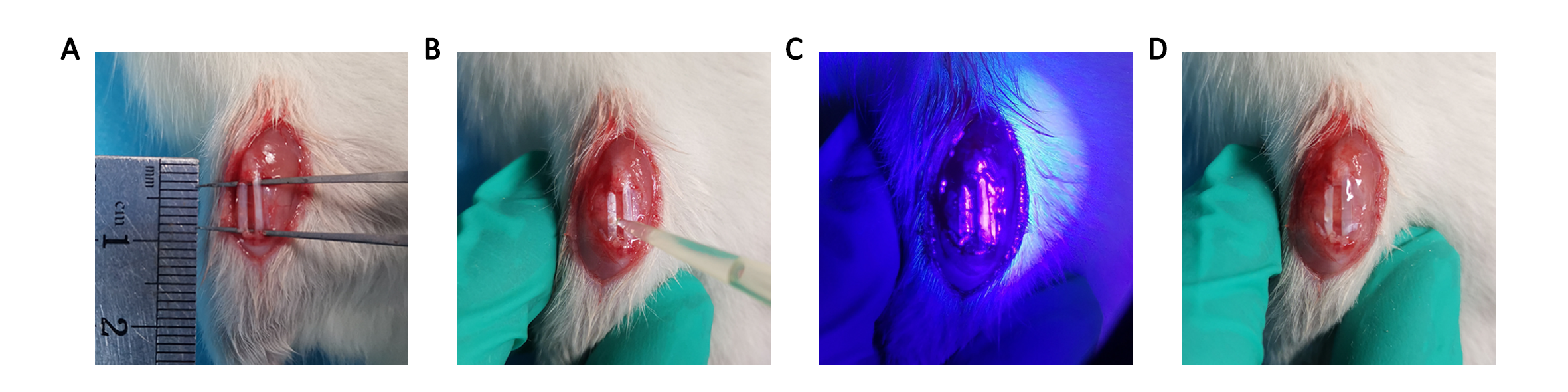

Supplement: Supplementary file 1 — Additional file 1: Figure S1. Patellar tendon injury model. A Exposure and removal of central 1/3 right patellar tendon tissue. B ADSC-Exos-loaded GelMA injected into the location of the patellar tendon defect. C Radiation GelMA with 405nm light source for 30s. D Photocross-linking under radiation forms a gel state. [file 13287_2021_2410_MOESM1_ESM.tif]

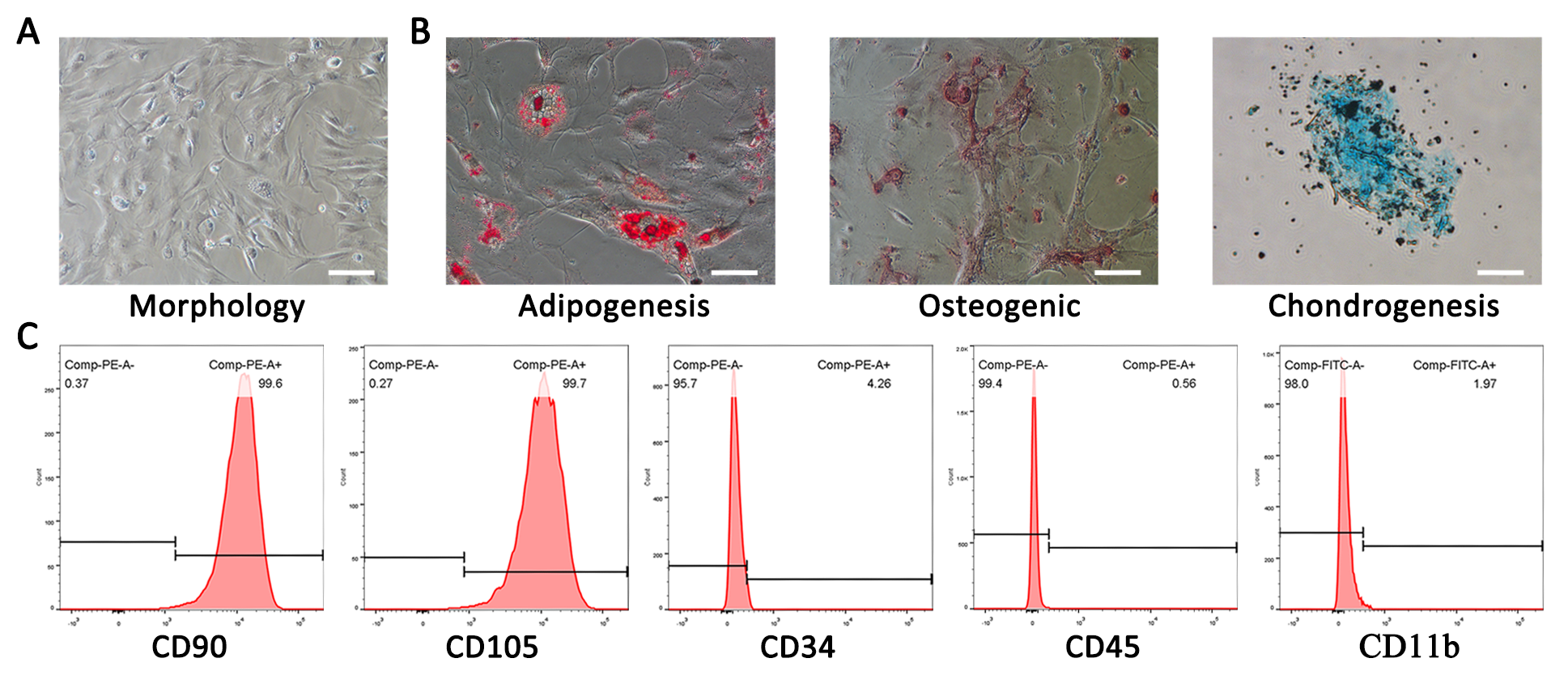

Supplement: Supplementary file 2 — Additional file 2: Figure S2. Characterization of ADSCs. A Morphology of ADSCs. B Adipogenic, osteogenic and chondrogenic differentiation of ADSCs. C Flow cytometry for detection of ADSC surface markers. Bars, 100μm. [file 13287_2021_2410_MOESM2_ESM.tif]
